# Supplementary material for: Pyruvate Dehydrogenase Kinase 1 inhibition mediated oxidative phosphorylation enhancement in cartilage promotes osteoarthritis progression
Source: BMC Musculoskelet Disord. 2023 Jul 20;24:597. doi: 10.1186/s12891-023-06585-6 (PMC10357736; doi:10.1186/s12891-023-06585-6)
Supplement: Supplementary file 1 — Additional file 1. [file 12891_2023_6585_MOESM1_ESM.zip › MF.Gsea.1653627316242/GOMF_DNA_REPLICATION_ORIGIN_BINDING.html]

Details for gene set GOMF\_DNA\_REPLICATION\_ORIGIN\_BINDING[GSEA]

|  || Dataset | 55457\_collapsed\_to\_symbols.cls.cls #OA\_versus\_normal.cls.cls #OA\_versus\_normal\_repos |
| Phenotype | cls.cls#OA\_versus\_normal\_repos |
| Upregulated in class | OA |
| GeneSet | GOMF\_DNA\_REPLICATION\_ORIGIN\_BINDING |
| Enrichment Score (ES) | 0.44308627 |
| Normalized Enrichment Score (NES) | 1.370904 |
| Nominal p-value | 0.08783784 |
| FDR q-value | 0.3514805 |
| FWER p-Value | 1.0 |
Table: GSEA Results Summary

  

Fig 1: Enrichment plot: GOMF\_DNA\_REPLICATION\_ORIGIN\_BINDING      
 Profile of the Running ES Score & Positions of GeneSet Members on the Rank Ordered List

  

| SYMBOL | TITLE | RANK IN GENE LIST | RANK METRIC SCORE | RUNNING ES | CORE ENRICHMENT || 1 | MCM5 | minichromosome maintenance complex component 5 [Source:HGNC Symbol;Acc:HGNC:6948] | 351 | 0.724 | 0.0655 | Yes |
| 2 | ORC5 | origin recognition complex subunit 5 [Source:HGNC Symbol;Acc:HGNC:8491] | 422 | 0.697 | 0.1493 | Yes |
| 3 | MCM2 | minichromosome maintenance complex component 2 [Source:HGNC Symbol;Acc:HGNC:6944] | 888 | 0.546 | 0.1832 | Yes |
| 4 | KAT7 | lysine acetyltransferase 7 [Source:HGNC Symbol;Acc:HGNC:17016] | 1125 | 0.491 | 0.2278 | Yes |
| 5 | ORC3 | origin recognition complex subunit 3 [Source:HGNC Symbol;Acc:HGNC:8489] | 1148 | 0.487 | 0.2885 | Yes |
| 6 | ORC4 | origin recognition complex subunit 4 [Source:HGNC Symbol;Acc:HGNC:8490] | 1234 | 0.470 | 0.3422 | Yes |
| 7 | CDC6 | cell division cycle 6 [Source:HGNC Symbol;Acc:HGNC:1744] | 1341 | 0.451 | 0.3917 | Yes |
| 8 | MCM10 | minichromosome maintenance 10 replication initiation factor [Source:HGNC Symbol;Acc:HGNC:18043] | 1410 | 0.442 | 0.4431 | Yes |
| 9 | POLA1 | "DNA polymerase alpha 1, catalytic subunit [Source:HGNC Symbol;Acc:HGNC:9173]" | 2300 | 0.327 | 0.4160 | No |
| 10 | ORC1 | origin recognition complex subunit 1 [Source:HGNC Symbol;Acc:HGNC:8487] | 2525 | 0.300 | 0.4370 | No |
| 11 | CDC45 | cell division cycle 45 [Source:HGNC Symbol;Acc:HGNC:1739] | 9982 | -0.319 | -0.1008 | No |
| 12 | ORC2 | origin recognition complex subunit 2 [Source:HGNC Symbol;Acc:HGNC:8488] | 10191 | -0.340 | -0.0734 | No |
| 13 | DHX9 | DExH-box helicase 9 [Source:HGNC Symbol;Acc:HGNC:2750] | 10627 | -0.386 | -0.0578 | No |
| 14 | HSPD1 | heat shock protein family D (Hsp60) member 1 [Source:HGNC Symbol;Acc:HGNC:5261] | 11427 | -0.492 | -0.0568 | No |
| 15 | GRWD1 | glutamate rich WD repeat containing 1 [Source:HGNC Symbol;Acc:HGNC:21270] | 11694 | -0.531 | -0.0093 | No |
| 16 | DDX11 | DEAD/H-box helicase 11 [Source:HGNC Symbol;Acc:HGNC:2736] | 12607 | -0.803 | 0.0227 | No |
Table: GSEA details [plain text format]

  

Fig 2: GOMF\_DNA\_REPLICATION\_ORIGIN\_BINDING      
 Blue-Pink O' Gram in the Space of the Analyzed GeneSet

  

Fig 3: GOMF\_DNA\_REPLICATION\_ORIGIN\_BINDING: Random ES distribution      
 Gene set null distribution of ES for **GOMF\_DNA\_REPLICATION\_ORIGIN\_BINDING**
